# Targeted proteomics identifies liquid-biopsy signatures for extracapsular prostate cancer

**DOI:** 10.1038/ncomms11906

**Published:** 2016-06-28

**Authors:** Yunee Kim, Jouhyun Jeon, Salvador Mejia, Cindy Q Yao, Vladimir Ignatchenko, Julius O Nyalwidhe, Anthony O Gramolini, Raymond S Lance, Dean A Troyer, Richard R Drake, Paul C Boutros, O. John Semmes, Thomas Kislinger

**Affiliations:** 1 Department of Medical Biophysics, University of Toronto, Toronto, Ontario, Canada M5G 1L7; 2 Informatics and Bio-computing Program, Ontario Institute for Cancer Research, Toronto, Ontario, Canada M5G 0A3; 3 Princess Margaret Cancer Center, University Health Network, Toronto, Ontario, Canada M5G 1L7; 4 Department of Microbiology and Molecular Cell Biology, Eastern Virginia Medical School, Norfolk, Virginia 23507, USA; 5 Leroy T. Canoles Jr. Cancer Research Center, Eastern Virginia Medical School, Norfolk, Virginia 23507-1627, USA; 6 Department of Physiology, University of Toronto, Toronto, Ontario, Canada M5S 1A8; 7 Department of Urology, Eastern Virginia Medical School, Norfolk, Virginia 23462, USA; 8 Department of Cell and Molecular Pharmacology and Experimental Therapeutics, Medical University of South Carolina, Charleston, South Carolina 29425, USA; 9 Department of Pharmacology and Toxicology, University of Toronto, Toronto, Ontario, Canada M5S 1A8

## Abstract

Biomarkers are rapidly gaining importance in personalized medicine. Although numerous molecular signatures have been developed over the past decade, there is a lack of overlap and many biomarkers fail to validate in independent patient cohorts and hence are not useful for clinical application. For these reasons, identification of novel and robust biomarkers remains a formidable challenge. We combine targeted proteomics with computational biology to discover robust proteomic signatures for prostate cancer. Quantitative proteomics conducted in expressed prostatic secretions from men with extraprostatic and organ-confined prostate cancers identified 133 differentially expressed proteins. Using synthetic peptides, we evaluate them by targeted proteomics in a 74-patient cohort of expressed prostatic secretions in urine. We quantify a panel of 34 candidates in an independent 207-patient cohort. We apply machine-learning approaches to develop clinical predictive models for prostate cancer diagnosis and prognosis. Our results demonstrate that computationally guided proteomics can discover highly accurate non-invasive biomarkers.

## Abstract (web summary)

Proteomic technologies are capable of identifying thousands of proteins in biological samples, but biomarker applications are lagging. Here the authors use Multiple Reaction Monitoring Mass Spectrometry to delineate peptide signatures that accurately distinguish between defined prostate cancer patient risk groups.

## Graphical abstract

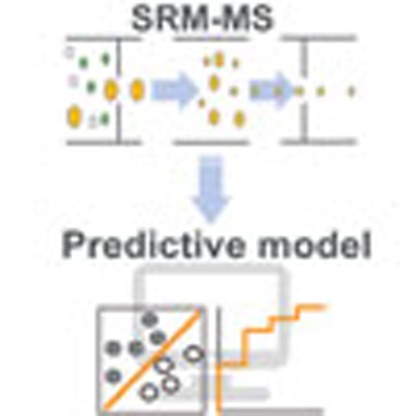

The worldwide incidence of prostate cancer has been steadily increasing, but many patients harbour tumours of an indolent nature. These indolent tumours grow slowly and pose minimal threat to the life of the patient, in the absence of treatment (that is, are clinically insignificant). However, once prostate cancer begins to grow aggressively, it metastasizes quickly with lethal consequences. The management of prostate cancer has become an urgent clinical dilemma with significant over-diagnosis and challenges in predicting patient survival^1^. Prostate cancers are uniquely heterogeneous with major spatial^2^,^3^ and temporal^4^ variability in their genomes. Therefore, once cancer has been confirmed, the optimal course of action is tailored to spare patients with indolent disease from unnecessary procedures, while identifying and treating those who would benefit from treatment intensification. Current clinical stratification employs the Gleason score (GS), estimates of tumour size and pretreatment prostate-specific antigen (PSA) levels. However, generating the GS requires obtaining biopsy specimens, a procedure that increases the risk of hospitalizations from post-biopsy complications, posing a significant burden on healthcare and risking serious complications^5^,^6^. In addition, biopsies under-sample the prostate, and significant lesions are frequently missed^7^. Localized prostate cancer has excellent prognosis, with almost 100% 5-year survival (http://seer.cancer.gov/statistics/summaries.html), but ∼70% of these men receive early intervention in the form of surgery or radiotherapy^8^,^9^,^10^,^11^,^12^. These therapies carry significant morbidities and healthcare costs, and surveillance protocols rely on repeat PSA testing, digital rectal examination (DRE) and multiple biopsies^13^,^14^. A major gap that currently exists in prostate cancer diagnostics is that no accurate biomarkers are available that could overcome these potential complications (that is, invasive and heterogeneity). A fluid-based biomarker would be ideal.

Liquid biopsies, such as circulating tumour cells^15^ and cell-free DNA^16^, have been proposed as promising non-invasive prostate cancer biomarkers, but their detection and enrichment remain technically challenging. Cataloguing the secreted and soluble factors released into the interstitial fluid that bathes the organ of interest may provide a novel inventory of putative disease biomarkers. We have interrogated the collection of proteins comprising a prostate-proximal fluid, expressed prostatic secretions (EPSs)^17^,^18^,^19^,^20^, that is collected either directly from the prostate before radical prostatectomy (termed: direct EPS) or from post-DRE urine (termed: post-DRE urine or EPS urine). Reproducible detection and quantification of multiple proteins in complex biological matrices is an essential requirement for any potential disease biomarker, but verification of these candidates is a major bottleneck in the pipeline from discovery to clinical implementation. Traditionally, immunoaffinity-based assays, namely, enzyme-linked immunosorbent assays are used to validate protein biomarkers, but this approach is time-consuming, costly and relies on the existence of validated antibody pairs for every target protein. Targeted mass spectrometry (MS) offers an alternative approach for the rapid verification of candidate biomarkers. Selected reaction monitoring mass spectrometry (SRM-MS) is currently the leading method for targeted quantification of proteins via MS. It offers excellent selectivity, sensitivity and throughput, without the need for validated antibodies.

Here we build on our proteomics studies of EPS and systematically develop SRM-MS assays in post-DRE urine samples. Step-wise applications of these assays to two independent, richly annotated patient cohorts in conjunction with computational modelling identify liquid-biopsy signatures that accurately distinguish patients with organ-confined (OC) stage pT2 and extracapsular stage pT3 prostate cancers, before radical prostatectomy. These data highlight the value of readily accessible tissue proximal fluids and multiplexed quantitative proteomic signatures to identify extracapsular disease before invasive surgery, potentially modifying the treatment options for these patients.

## Results

### Candidate selection and assay evaluation

**Figure 1:**
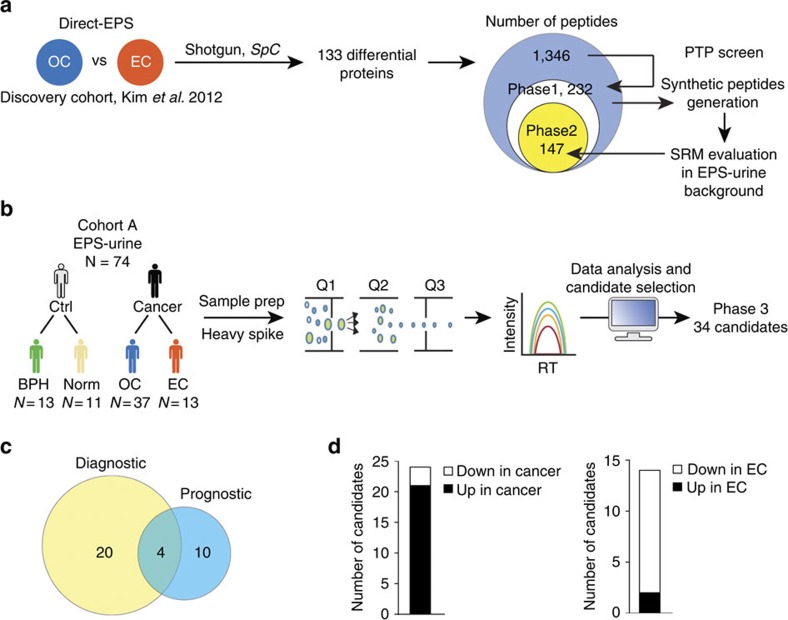
Systematic development of targeted proteomics assays in EPS urine samples. (**a**) Discovery proteomics data from direct EPS derived from patients with extracapsular (EC) or organ-confined (OC) prostatic tumours was used to select putative candidates. Proteotypic peptides from these candidates were carefully selected and evaluated by SRM-MS in an EPS urine background. (**b**) All peptides that passed the above selection criteria were analysed in clinically stratified EPS urine samples (cohort A). Peptide quantification by SRM-MS was performed, and 34 candidates with diagnostic and prognostic potential were identified based on the relative abundance changes. (**c**) Venn diagram depicting the distribution of peptides with diagnostic potential (that is, differential expression in cancer versus controls) and the number of peptides with prognostic potential (that is, differential expression in EC versus OC tumours). (**d**) Bar charts depict directional expression of the 34 peptide candidates; diagnostic—left panel, prognostic—right panel. PTP, proteotypic peptides; SpC, spectral counts. Pictograms adapted from vector files by Dave, http://vector4free.com/vector/man-woman-sign-pictograms/ (CC BY 4.0).

Our previously generated discovery proteomic profiles from direct EPS derived from extracapsular (EC) or OC prostate cancers laid the foundation for the current project^18^. Relative quantification revealed 133 proteins that were significantly differentially expressed between both patient groups. These proteins were identified by 1,346 distinct peptide sequences. Data mining of all peptides based on sequence and biophysical properties led to 232 proteotypic peptides (phase 1 peptides) suitable for evaluation by SRM-MS in EPS urine samples (Fig. 1a). To abrogate potential confounding influences due to the differing modes of data acquisition (discovery proteomics versus targeted proteomics), instruments (ion trap versus triple quadrupole) and sample types (direct EPS versus EPS urine), all phase 1 peptides were first evaluated for reproducible detection by SRM-MS in EPS urine samples. Each phase 1 peptide was purchased as a crude heavy-isotope-labelled synthetic peptide and spiked into pooled EPS urine samples to evaluate their suitability for targeted proteomics assays, directly within the biomarker matrix. Light (endogenous) and heavy (synthetic) peptides were monitored in SRM mode, and data were manually inspected to select peptides that had at least three fragment ions aligned at the expected peptide elution time, had co-eluting light and heavy peptides, had minimal interference and were reproducible. In total, 147 peptides (63%) met these quality criteria (phase 2 peptides), and were taken forward to an independent cohort of EPS urine samples (Fig. 1a). These results demonstrate that systematic SRM-MS assay development based on previous discovery proteomics data is rapid and feasible in clinically useful EPS urine samples.

### Peptide quantification in EPS urine cohort A

To evaluate phase 2 peptides, we performed relative quantification in a medium-sized cohort of EPS urine samples (*n*=74; cohort A; Table 1), using the crude heavy-isotope-labelled synthetic peptides as internal standards (Methods). The goal of this initial quantification was to evaluate peptide performance in relevant clinical samples, while reducing the number of peptides to be moved forward to the next development steps. Briefly, a Student's *t*-test was performed to compare the ratios of peptide abundance between cancer and non-cancer groups (termed: diagnostic), as well as EC and OC prostate cancers (termed: prognostic). The first criteria used to select candidates as potential diagnostic and prognostic biomarkers were *P* value cutoff points of 0.05 and 0.1, respectively. A higher *P* value cutoff was used to select prognostic candidates to avoid removing putative candidates at this early stage for distinguishing cancer comparisons (EC versus OC cancer groups). Refinements to the candidate list were made by adding peptides representing the proteins *IGJ* and *ANXA1*, because these peptides were the only candidates even trend upregulated in the EC tumour group (*P*≤0.25). Furthermore, two *KLK3* peptides were added to monitor PSA levels. Finally, each peptide that met the above criteria was manually inspected for SRM-MS trace quality. Overall, 34 candidates (phase 3 peptides) demonstrated a potential for classifying individuals based on the prostatic disease status (Fig. 1b). Overall, 24 diagnostic candidates (21 overexpressed in cancers) and 14 prognostic ones (2 overexpressed in invasive tumours) were identified (Fig. 1c,d). Our data suggest that SRM-MS assays developed in EPS urine samples are capable of separating patients into defined categories using retrospective cohorts, while quickly triaging candidates affected by large patient variability.

### Absolute quantification in EPS urine cohort B

**Figure 2:**
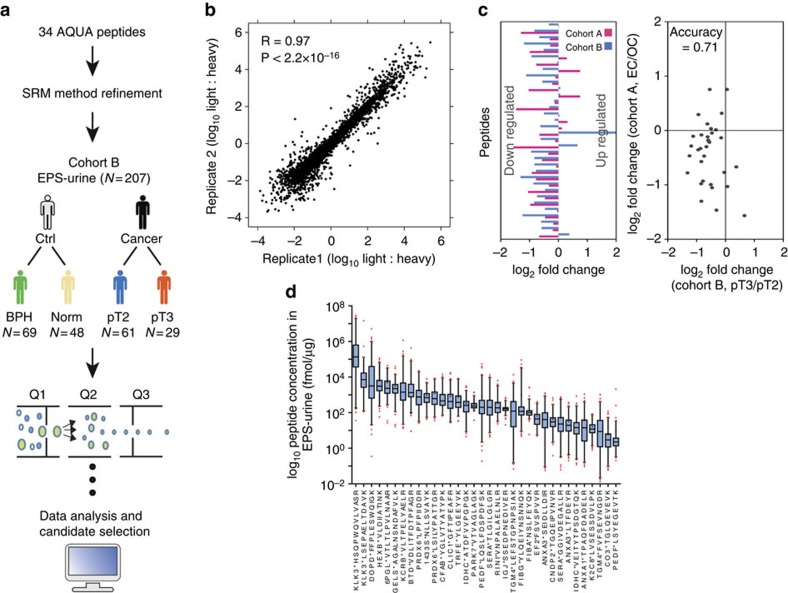
Absolute peptide quantification in an independent patient cohort. (**a**) All 34 peptide candidates were accurately quantified in an independent cohort of EPS urine samples (cohort B). (**b**) Peptide abundance correlation between both replicates analysed by SRM-MS. (**c**) Correlation of peptide expression for all 34 peptides in EPS urine samples from cohort A and B (prognostic comparison shown). Left panel: directionality of peptide expression; right panel: correlation plot. (**d**) Absolute peptide quantification in all EPS urine samples from cohort B. Box plots represent the median and interquartile range. Whiskers represent the 1–99 percentile. Outliers are represented by red dots and the mean is represented by ‘+'. Pictograms adapted from vector files by Dave, http://vector4free.com/vector/man-woman-sign-pictograms/ (CC BY 4.0).

To generate optimized SRM-MS assays for absolute quantification, highly purified (>97% purity) heavy stable isotope-labelled standards (AQUA peptides) were purchased for all 34 phase 3 peptides. Before quantification in independent samples (*n*=207; cohort B, Table 1), a multiplexed, scheduled SRM-MS method was developed, enabling quantification of all candidate peptides in a single chromatographic gradient (Fig. 2a; Methods). Next, all phase 3 peptides were quantified, using single-point quantification based on 100 fmol addition of each AQUA peptide to standardized amounts of total EPS urine (1 μg total protein on column), in an independent cohort of 207 EPS urine samples (Table 1; Methods). Each EPS urine was measured in two technical replicates. The duplicate analyses demonstrated strong reproducibility with a Pearson's correlation coefficient (*R*) of 0.97 (*P*<2.2 × 10^−16^; Fig. 2b). Of the 34 peptides, the majority (30 peptides, 88.24%) showed strong correlations between two replicates (*R*>0.7; Supplementary Fig. 1). Furthermore, we examined the reproducibility depending on sample types (normal, benign prostatic hyperplasia (BPH), pT2 and pT3 samples) and found that 31 peptides (91.18%) show strong reproducibility (*R*>0.7) in at least one type of sample (Supplementary Fig. 2). Peptides with *R*≤0.7 were generally of lower abundance (mean difference of peptide abundance (log_2_ L:H ratio) between the groups is 4.96, *P*=0.01, Student's *t*-test; Supplementary Fig. 3). The analysis also demonstrated good chromatographic reproducibility (coefficient of variation<1.5%) with most peptides eluting between 20 and 30 min over the 40-min chromatographic gradient (Supplementary Fig. 4a,b).

To compare the correlation of our quantitative results in both EPS urine cohorts, fold-change correlation plots were generated for the various group comparisons. While different EPS urine cohorts, spike-in standards and SRM-MS methods were used, we were able to observe a generally positive correlation. On average, 73% of our peptides showed concordant expression in both cohorts (Fig. 2c; Supplementary Fig. 5).

**Figure 3:**
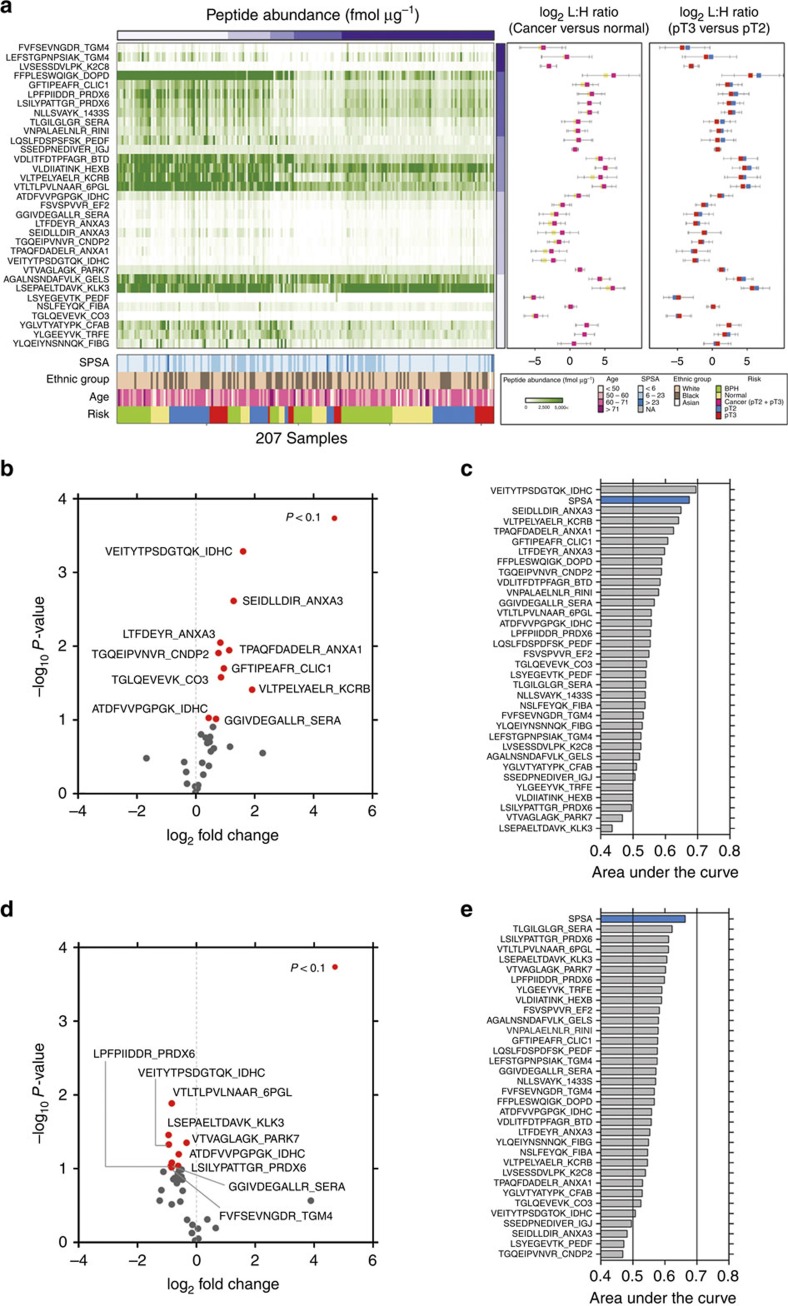
Univariate analyses to distinguish patient risk groups. (**a**) Heatmap representation of absolute peptide expression levels for all candidate peptides within cohort B samples (represented as fmol μg^−1^ EPS urine protein). Peptide expression heatmap is clustered using consensus clustering. Pearson's correlation was used as the similarity metric to generate clusters and k-means method (*k*=5) was used as a clustering algorithm. Serum PSA (SPSA) levels, ethnicity and patient risk group status are shown. On the right-hand side peptide expression levels for ‘cancer versus normal' and ‘pT3 versus pT2' prostate cancers are represented as box plots (shown as log_2_ ratios of endogenous ‘L' divided by spike-in standard ‘H' peak ratios). (**b**) Quantification of individual peptides in normal versus all cancer patient EPS urine samples. Peptides passing indicated statistical cutoff criteria are colour-coded in red. Peptide sequences and gene names are indicated. (**c**) The area under the ROC curve (AUC) was used to evaluate the ability of individual peptides to distinguish between cancer patients and normal controls. SPSA values, available for these patients, were used as a positive control (indicated as blue bar). (**d**,**e**) Same analyses performed for prostate cancer risk groups (pT3 versus pT2).

Next, the absolute concentration of each peptide across all 207 samples in cohort B was determined. The 34 peptides quantified spanned approximately five orders of magnitude, from the low fmol to nmol range per μg total EPS urine protein (Fig. 2d; ref. 21). As expected, the two most abundant peptides represented *KLK3*, and in fact the *KLK3* peptide HSQPWQVLVASR demonstrated such a strong detector response that it was ultimately removed from further analyses, as it would require sample dilution to achieve accurate quantification via AQUA spike-in. The remaining peptides spanned approximately three orders of magnitude and significant inter-patient variation in peptide expression was observed (Fig. 3a). These data demonstrate that selected biomarker candidates can be rapidly quantified in large numbers of EPS urine samples spanning approximately five orders of concentration.

### Univariate analyses to distinguish patient groups

We investigated the abundance of peptides in different patient risk groups (normal, BPH, pT2 and pT3). All risk groups had similar age distributions (average age of 60 years) and ethnic compositions (White American=62%, Black American=38% and Asian American=0.5%; Fig. 3a; Supplementary Fig. 6a,b). As we expected, normal controls had the lowest concentration of serum PSA (SPSA, average 5.06 ng ml^−1^) and pT3 stage had the highest concentration of SPSA (average 7.60 ng ml^−1^, *P*=0.03, Student's *t*-test; Supplementary Fig. 6c). Next, we evaluated the ability of individual peptides to distinguish among distinct patient groups based on the absolute quantification of individual peptides. Most (27/33) are more abundant in cancer patients (middle panel in Fig. 3a) compared with normal controls (1.03–4.82 times more expressed; Fig. 3b). Among them, 10 peptides had, on average, 2.2-fold change in expression (1.4–3.8-fold changes) with *P*<0.1 (Student's *t*-test, red dots in Fig. 3b). Further, of all quantified peptides, 28 (84.85%) were underexpressed in pT3 stage (right panel in Fig. 3a) compared with pT2 stage (1.03–2.37 times underexpressed; Fig. 3d). Nine peptides were significantly downregulated in pT3 stage (0.5–0.8-fold changes with *P*<0.1, Student's *t*-test; red dots in Fig. 3d).

To evaluate the power of each peptide to distinguish individual patient risk groups, we measured the area under the receiver operating characteristic curve (AUC) of each peptide. For diagnosis (cancer versus normal), one peptide (VEITYTPSDGTQK) showed higher AUC (AUC=0.69) than SPSA (AUC=0.67), the currently used biomarker (Fig. 3c). For prognosis (pT3 versus pT2), SPSA showed the highest prediction ability (AUC=0.66, Fig. 3e). Neither SPSA nor any of our individually selected peptides were able to accurately predict patient risk groups with high confidence (AUC>0.7). These findings are in line with the general belief that signatures, rather than individual biomarkers, are required to accurately distinguish patient groups.

### Signatures that distinguish patient groups

**Figure 4:**
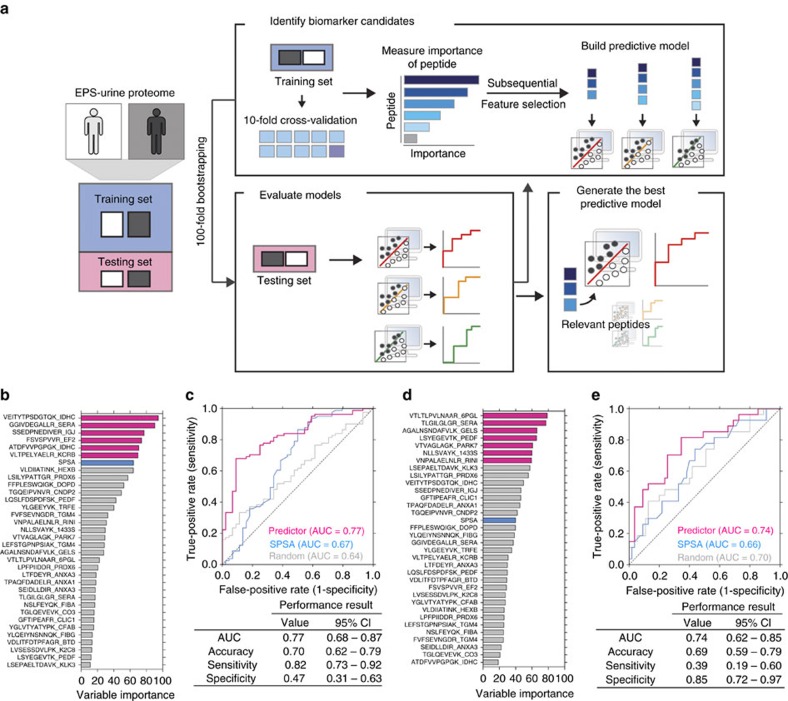
Machine-learning model to identify biomarker signatures. (**a**) Schematic overview of the machine-learning approach used to develop multi-feature biomarker signatures. (**b**) The predictive importance of individual peptides to distinguish prostate cancer from normal controls. Pink bars represent the selected relevant peptides to build the predictor. Blue bar represents the predictive importance of serum PSA (SPSA). (**c**) ROC curves for diagnosis. The performance for the selected peptide signature (pink), SPSA alone (blue) and randomly selected peptides (grey) are compared. ROC curves are generated from 10-fold cross-validation. ROC curves generated from test set are in Supplementary Fig. 7. (**d**) The predictive importance of individual peptides to distinguish pathological stage pT3 from stage pT2. (**e**) ROC curve analyses for prognosis. Pictograms adapted from vector files by Dave, http://vector4free.com/vector/man-woman-sign-pictograms/ (CC BY 4.0).

To identify subsets of peptides that can serve as liquid-biopsy signatures and integrate them into unified predictors that accurately discriminate among our distinct patient risk groups, we employed a machine-learning analysis (Fig. 4a). Briefly, we evaluated quantified peptides as input features for machine learning and identified the most relevant ones using Generalized Linear Models (GLMs)^22^. The selection of reliable features reduces the dimensionality of the feature space and leads to better performance in machine learning^23^. To select relevant peptides, we measured the importance of each peptide to discriminate two classes of patient risk groups (cancer versus normal controls or pT2 versus pT3 prostate cancers) and subsequently systematically selected the top-ranked peptides as features to build predictive models. To obtain stable predictions, predictive models were tested by 100-fold bootstrapping (Methods). We evaluated the prediction performance of selected peptides using AUC. A set of peptides that showed the highest AUC was selected as the most relevant features. Using these peptides as features, the best predictive model was generated.

We found that the six top-ranked features, mapping to five distinct gene products (*IDHC*, *SERA*, *IGJ*, *EF2* and *KCRB*; pink bars in Fig. 4b) showed the best performance to discriminate all prostate cancer patients (pT2 and pT3) from biopsy-verified normal controls. From the 10-fold cross-validation, these features predicted 70% of cases correctly (82% sensitivity and 47% specificity). We found that a combination of these peptides outperforms the traditional Food and Drug Administration-approved biomarker, PSA. The predictive model showed an AUC of 0.77 (95% confidence interval, 0.68–0.87, pink line in Fig. 4c). In the test set (10% of entire data set), our predictive model showed an AUC of 0.79 (95% of confidence interval, 0.76–0.81), 82% sensitivity and 49% specificity (Supplementary Fig. 7a). This compares favourably with the performance of SPSA alone in which the AUC is 0.67 (blue line in Fig. 4c), similar to previous reports^24^. Furthermore, we compared our six-peptide biomarkers to the null distribution in this data set by performing a large-scale re-sampling study^1^,^25^. We generated 100 random sets of six peptides, trained them using the same GLM approach used above and measured the performance using AUC. A random model achieved an AUC of 0.64 from the 10-fold cross-validation (grey line in Fig. 4c) and 0.61 from test set (Supplementary Fig. 7a). Indeed, our selected peptide biomarkers showed a significant improvement with respect to randomly selected signatures (bootstrap *P*=3.33 × 10^−32^; Supplementary Fig. 8). Taken together, targeted proteomics quantification in post-DRE urine samples is capable of identifying peptide signatures that are superior to the current gold standard biomarker for prostate cancer screening.

We next sought to examine whether our approach can be used to address an important clinical challenge: to distinguish pT2 stage (OC) tumours from pT3 stage (EC) tumours, before radical prostatectomy. Of note, our retrospective patient cohort B had detailed clinical annotation, both at the stage of needle biopsy and following surgical resection of the prostate (Supplementary Table 1). It is hence important to note that all patients' tumours were staged as T2 via diagnostic needle biopsy, but up-staged to pT3 following precise pathological examination of the surgical specimen. From 100-fold bootstrapping, we selected seven top-ranked peptides (*6PGL*, *SERA*, *GELS*, *PEDF*, *PARK7*, *1433S* and *RINI*; pink bars in Fig. 4d) as the most relevant biomarker signatures to predict pT3 samples before radical prostatectomy (that is, EPS urine samples were collected before surgery). From the 10-fold cross-validation, the combination of these seven features showed AUC of 0.74 (95% confidence interval, 0.62–0.85), 69% accuracy, 39% sensitivity and 85% specificity (Fig. 4e). In the test set, our predictive model achieved 74% accuracy and an AUC of 0.77 (95% confidence interval, 0.74–0.80; Supplementary Fig. 7b). Meanwhile, the single SPSA-based approach achieved an AUC of 0.66 and a random model achieved an AUC of 0.70 (10-fold cross–validation; Fig. 4e) and 0.69 (test set, Supplementary Fig. 7b). Our predictive model significantly outperformed 100 random sets of seven peptide signatures (bootstrap *P*=4.57 × 10^−7^; Supplementary Fig. 8). These results suggest that targeted proteomics analyses in clinically relevant tissue proximal fluids are capable of detecting peptide signatures that predict prostate cancer stage independent of needle biopsy and before surgical removal of the prostate.

## Discussion

The timely verification of extensive lists of candidate disease biomarkers generated by high-resolution proteomic technologies is becoming increasingly feasible and necessary to identify putative candidates. Despite this, the implementation of biomarkers into clinical practice is largely lagging behind. This is, in part, attributed to the lack of validated methods of candidate testing and further evaluation. Targeted proteomics by SRM-MS has emerged as the method of choice for candidate protein quantification and verification, due to its relatively low cost and amenability to robust high-throughput assay development workflows^26^,^27^,^28^. The aim of the current study was to systematically evaluate targeted proteomic assays in a clinically applicable, yet to date rarely utilized tissue proximal fluid—EPS urine—and develop liquid-biopsy signatures for accurate patient classification.

This work is an extension of previously published proteomics data in direct EPS from patients with EC and OC tumours^18^. The feasibility of obtaining coordinates (peptide elution times and the most intense fragment ions) from different modes of data acquisition and instrumentation was demonstrated by extracting relevant information from shotgun proteomics to lay the foundation for targeted assay development using a triple quadrupole mass spectrometer. By performing multiple rounds of assay refinement and statistical evaluation, we narrowed over 200 promising candidates to 34 peptides with high biomarker potential. Using heavy-isotope-labelled peptide standards, we estimated and later absolutely quantified these candidates in richly annotated cohorts of EPS urine samples.

Interestingly, a small number of peptides in EPS urine overlapped with those that were previously explored in urine^29^. The cancer-associated proteins that were evaluated in that study were empirically gathered from reports based on protein and nucleic-acid changes in human plasma and tissue^29^,^30^. In contrast, we directly compared prostate-proximal fluids from EC and OC tumour groups to derive biomarker candidates. We previously demonstrated that post-DRE urine samples (EPS urine samples) contain a unique subset of proteins, when compared with matched urine samples from the same patient, supporting our hypothesis that prostate-enriched proteins are released as a result of the DRE^19^. Interestingly, applying a subset of our SRM-MS assays to an independent cohort of prostate cancer urine samples (non post-DRE urine samples) demonstrated that the majority of peptides were not quantifiable and likely below our detection limits.

A notable finding from the work presented here is the trend towards lower abundance of the majority of candidates with advancing disease. For instance, elevated serum levels of PSA are indicative of prostate cancer; however, EPS levels of PSA have been consistently lower in disease involving EC extension (EC and/or pT3)^18^. Similarly, a trend of decreased PSA in EPS urine from cancer patients compared with controls has been noted by Drake *et al.*^31^. This may be indicative of PSA leakage out of the prostate gland and into the circulation^32^ or by diminished secretory functions that have been observed for high GS tumours, a histology hallmark of which are smaller, rounder glands. Similarly, it can be conceptualized that other proteins are escaping the prostate and entering the circulation due to the deteriorating structural integrity of advanced prostate cancers. It would be beneficial to measure both EPS and serum levels of such proteins from the same patient to further test this observation. Although the majority of candidate expression levels were in agreement between cohorts A and B, some differences were noted. For instance, the protein *ANXA3* has one peptide (LTFDEYR) that is upregulated in the EC group of EPS urine samples in cohort A, while the other peptide (SEIDLLDIR) is downregulated in the EC group. This could be due to inaccurate quantification, the presence of multiple proteoforms^33^, post-translational modifications or variations in proteolytic digestion efficiencies of endogenous proteins^34^,^35^ and warrant further investigation using additional peptides.

To evaluate the effect of extra peptides from the same protein on risk assessment, we examined the performance of our predictors by adding these additional peptides. In cohort B, there are six proteins (*ANXA3*, *IDHC*, *PEDF*, *PRDX6*, *SERA* and *TGM4*) that each have two unique peptides used in our SRM-MS assays. Our original predictive models contained both *IDHC* peptides and one *SERA* peptide (diagnostic model) and one *SERA* peptide (prognostic model). We hence evaluated if adding the second *SERA* peptide would change the performance of our predictors. As it turns out, there is no significant performance change between our original predictors (AUC of 0.79 for diagnosis and 0.77 for prognosis) and the modified prediction models using both peptides (AUC of 0.81 for diagnosis with *P* value=0.18, AUC of 0.77 for prognosis with *P* value=0.97, Student's *t*-test; Supplementary Table 2). Indeed, the additional SERA peptide has a low variable importance score. For example, for the diagnostic analysis, the additional peptide ‘TLGILGLGR' from *SERA* has a variable importance score of 17.04. Meanwhile, the selected peptide ‘GGIVDEGALLR' from *SERA* has a variable importance score of 90.30 (average variable importance score is 37.21; Fig. 4b).

For the comparison between BPH and normal individuals from cohort B, only the protein *IDHC* (peptide VEITYTPSDGTQK) was found to demonstrate a significant increase in BPH. This may be explained by the fact that the original discovery cohort^18^ lacked a non-cancer group consisting of BPH and normal individuals. Furthermore, candidates who were selected from cohort A for verification were not specifically aimed at verifying their differential expression between BPH and normal EPS urine samples. Future studies could be aimed at identification of peptide signatures that accurately distinguish normal from BPH, since this is a major limitation of SPSA measurements.

Although not obtained for the current study, additional parameters such as tumour microenvironment measurements may also provide information about patient outcome. Indeed, in a recent study, intra-prostatic hypoxia was combined with DNA indices (copy-number alterations) to robustly predict 5-year biochemical recurrence (BCR)^1^. Such multi-feature signatures may provide a comprehensive, powerful predictor of patient outcome. Patients enrolled in cohort B of this study have clinical information regarding BCR. In the pT2 group, 11 individuals out of 61 developed BCR within 2 years post RP; in the pT3 group, 10 individuals out of 29 developed BCR. Although not yet explored in this study, one approach to the analysis of the data may be to comparatively analyse the protein expression changes between individuals who developed recurrence and those that did not.

Our study is currently the largest investigation of prostate-proximal fluids in the context of biomarker discovery utilizing discovery proteomic data to design targeted proteomic assays. While developed assays are subsequently evaluated across multiple independent cohorts, substantial work is still required before possible clinical application. This will include additional validation in independent patient cohorts, preferably using longitudinally collected samples, and additional assay optimizations^36^. Furthermore, alternative approaches such as analyses of extracellular vesicles^37^ or glycosylated proteins^38^,^39^ should be applied for the discovery of prostate cancer biomarkers, ideally in parallel to analyses of matching tissue specimens applying carefully designed multi-omic/proteogenomic workflows^40^. Here we provide the first work that utilizes SRM-MS for the systematic identification of novel biomarker signatures for distinguishing prostate cancer patient risk groups in a medium-sized cohort of biofluid.

## Methods

### Sample collection and annotation

Samples were obtained from patients following informed consent and use of Institutional Review Board approved protocols at Urology of Virginia and Eastern Virginia Medical School (#06–12-FB-0343) and the Research Ethics Review Board at the University Health Network (10–0159-T). A measure of 20 ml of EPS urine was centrifuged at 1,100*g* for 15 min at 4 °C to pellet debris. The resulting EPS urine supernatant was aliquoted in volumes of 3.5 ml and diluted with 2.5 ml of PBS (pH 7.4). The mixture was vortexed, combined and centrifuged at 1,100*g* for 15 min. The resulting supernatants were stored at −40 °C. Detailed clinical information for all patients enrolled in this study are available (Supplementary Table 1). Prostate cancer patients were selected on the basis of organ confinement or pathological stage. Non-cancer individuals had biopsy-confirmed BPH or were considered as individuals with no indication of prostatic disease based on the biopsy results.

### Sample preparation for MS

Ultrapure-grade 2,2,2-trifluoroethanol, trifluoroacetic acid, iodoacetamide and dithiotreitol (DTT) were from Sigma-Aldrich. High-performance liquid chromatography-grade solvents (methanol, acetonitrile and water) and formic acid were from Fisher Scientific. MS-grade trypsin/Lys-C was from Promega (Madison, WI). Amicon spin filters, 0.5 ml, 3 kDa molecular weight cut-off (MWCO), were from Millipore. Solid-phase extraction C18 tips were from Agilent. A measure of 4 ml of EPS urine was concentrated to ∼500 μl using a spin filter with a molecular weight cutoff of 3 kDa, and proteins were precipitated overnight by the addition of ice-cold 100% methanol. Protein pellets were washed twice with 100% methanol and air-dried. Protein resolubilization was performed by the addition of 50% 2,2,2-trifluoroethanol at 60 °C for 2 h. Following reduction with DTT and alkylation with iodoacetamide, proteins were digested overnight at 37 °C using 2 μg trypsin/Lys-C. The reaction was quenched by the addition of trifluoroacetic acid. Desalting was performed by solid-phase extraction using C18 tips. Solvents were removed by vacuum centrifugation, and peptides were resolubilized in 5% acetonitrile and 0.1% formic acid. Peptide concentrations were determined by the micro-BCA assay kit (Thermo Fisher Scientific).

### Selected reaction monitoring mass spectrometry

Samples were analysed on a TSQ Vantage triple quadrupole mass spectrometer (Thermo Fisher Scientific) equipped with an EASY-Spray (Thermo Fisher Scientific) electrospray ion source. Separations were performed on EASY-Spray columns (15 cm × 75 μm ID packed with 3-μm C18 particles, Thermo Fisher Scientific) heated to 50 °C. Peptides were kept at 4 °C and loaded onto the column from an EASY-nLC (Thermo Fisher Scientific) autosampler. Chromatographic conditions were as follows: 40-min gradient at a flow rate of 300 nl min^−1^ starting with 100% A (water), stepping up to 5% B (ACN) in 5 min, followed by 25% B at 35 min, followed by a steep increase to 50% B at 38 min and 100% B at 40 min. Targeted acquisition of eluting ions was performed by the mass spectrometer operated in SRM-MS mode with Q1 and Q3 set to 0.7 *m/z* full width at half maximum resolution and a cycle time of 1 s. For all SRM-MS runs, with the exception of the measurement of phase 3 peptides from cohort B, multiple unscheduled injections were used, each targeting ∼200 transitions. For cohort B, a single scheduled method was utilized with a 2-min elution window.

### Selection of phase 2 peptides

In a previous study^18^, 133 differentially expressed proteins were identified when comparing the proteome profiles of 16 direct EPS samples from individuals with EC and OC prostatic tumours. A spectral library was built from the resulting LTQ-Orbitrap XL data using the Skyline software tool (version 2.1.0)^41^. All spectra had scores that passed a stringent peptide score as determined by an X!Tandem target decoy search (∼0.5% false discovery rate). Protein sequences were converted to FASTA format and uploaded into Skyline for the prediction of proteotypic peptides. Peptides were chosen based on the previously reported specifications^42^. A total of 232 proteotypic peptides (phase 1 peptides) were selected and purchased as bulk heavy-isotope-labelled peptide standards (JPT Peptide Technologies), also containing eight peptides that were deemed potentially interesting from our additional EPS proteomics studies^17^,^19^,^20^. To assess the suitability of the phase 1 peptides for SRM-MS, 250 fmol of each heavy peptide standard was spiked into 1 μg of EPS urine digest, with four to six transitions monitored over a 40-min chromatographic gradient. Of the 232 phase 1 peptides, 147 (phase 2 peptides) were reproducibly detectable with a minimum of three transitions in the complex EPS urine background.

### Quantification of phase 2 and selection of phase 3 peptides

A cohort of individual EPS urine samples (*n*=74) from a heterogeneous population of patients with EC, OC and control (BPH and normal; cohort A) was used to analyse all phase 2 peptides. A total of 1 μg of peptide from each sample was spiked with 200 fmol of heavy peptide standards that were combined into six batches (batch A–F), consisting of ∼20 peptides per batch. Visualization and inspection of peaks were performed in Skyline. Each peptide was quantified in a sample by integrating the quantifier ion (most intense ion) of the light peptide with its co-eluting heavy peptide ion, to derive a light-to-heavy peptide ratio. The Student's *t*-test was used to compare the ratios between cancer and controls, as well as EC and OC prostate cancers. A K-fold cross-validation was performed to investigate the diagnostic and prognostic power of the peptides at different *P* value cutoff points. For the diagnostic and prognostic peptide candidates, *P* value cutoff points of 0.05 and 0.1 were used, respectively. Further refinements to this list were made by including additional peptides that did not meet the *P* value cutoff points, but were potentially promising. For instance, peptides SSEDPNEDIVER from protein *IGJ* and TPAQFDADELR from protein *ANXA1* were added to the list of putative prognostic candidates because they were the only candidates that were elevated in the EC tumour group (*P* value=0.25). Two *KLK3* peptides (HSQPWQVLVASR and LSEPAELTDAVK) were also added to monitor PSA levels in EPS urine. A total of 34 peptides, comprising the phase 3 candidates (24 diagnostic and 14 prognostic peptides, of which four overlapped), were taken forward for verification.

### Multiplexed SRM-MS

To increase throughput, a multiplexed SRM-MS assay was developed by scheduling all 34 candidates in a single 40-min chromatographic gradient. A total of three transitions were monitored for the light and heavy versions of each peptide for a total of 204 transitions per analysis. A 2-min acquisition time window was scheduled around the expected peptide elution time.

### Verification of phase 3 peptides in EPS urine samples

For verification, a heterogeneous population of patients with pathological stage pT3 and pT2 prostate tumours, BPH and normal individuals (*n*=207) were enrolled (cohort B). An amount of 1 μg of total peptide from each sample was spiked with 100 fmol of heavy peptide and 10 fmol of corresponding light peptide for all candidates with the exception of the *KLK3* peptide, HSQPWQVLVASR, which was spiked in at 500 fmol of heavy peptide. Visualization and inspection of peaks were performed on Skyline. Each sample was analysed in two technical replicates using the same instrument parameters as described above.

### Quantitative and statistical analyses

Each of the light and heavy peptides were checked for the quality of data by observing co-elution of all three transitions, alignment of light and heavy peptide elution times, and reproducibility between technical replicates. Relative ratios of the AUC of the most predominant ion (quantifier) of the light peptide versus the corresponding heavy quantifier were calculated. To evaluate the reproducibility of proteomics data, we compared peptide abundance in two replicates using Pearson's correlation coefficient (*R*). On average, peptides show the *R* of 0.97 across all samples. Of quantified peptides, 30 (88.24%), 28 (82.35%), 31 (91.18%) and 31 (91.18%) peptides show strong reproducibility (*R*>0.7) in normal controls, BPH, pT2 and pT3 samples, respectively (Supplementary Fig. 2). Peptides were comparatively tested for abundance differences between cancer versus normal and pathological stage pT3 versus pathological stage pT2. All statistical analyses were performed in R environment (v3.2.1).

### Generating predictors for prostate cancer

To generate a predictor that distinguishes cancer patients from control individuals (diagnosis), peptide expressions of 90 cancer patients (positive set) and 48 normal controls (negative set) were used. For the prediction of EC and OC cancers (prognosis), 29 pT3 samples (positive set) and 61 pT2 samples (negative set) were used (Supplementary Table 3). All data sets (positive and negative sets) were divided into two groups to build predictive models: a training set (90% of data set) and a testing set (10% of data set). When 90% of the data set was used as a training set, the predictive model was able to capture the properties of context classification associated with patient risk groups and, thus, showed the maximum performance on the testing set compared with other sized training sets (60% of data set and 80% of data set; Supplementary Table 4). To examine the reliability of sample size for the training, power analyses were performed (Supplementary Fig. 9). Power analyses allow for the determination of the appropriate sample size for statistical analysis. In general, there is a large difference effect between the two groups when effect size is bigger than 0.5 at a power of 0.8 (ref. 43). In our study, at a power of 0.8, the effect sizes of the training sets are 0.79 (diagnosis, cancer versus normal control, *P*=0.001) and 1.01 (prognosis, pT3 versus pT2, *P*=0.001). Power analysis was performed using ‘pwr' package (1.1–3) in R (version 3.2.1).

### Machine learning and feature selection

The GLM was used to classify samples into two classes: cancer versus normal (diagnosis) and pT3 versus pT2 (prognosis). GLM is a widely used machine-learning algorithm that has been applied in various types of biomarker identification (for example, cancer, HIV/AIDS and infection diseases) with reliable performance^44^,^45^,^46^. In addition, we demonstrate that GLM outperformed eight other machine-learning algorithms, which look for different types of patterns and data properties; *random forest* (rf), *stochastic gradient boosting* (gbm), *Naive Bayes* (nb), *boosted generalized linear model* (glmboost), *lasso and elastic-net-regularized generalized linear model* (glmnet), *support vector machine with linear kernel* (svmLinear) and *radial basis function (RBF) kernel* (svmRadial). We generated predictive models using all peptides, tested them by 100 bootstrap samples and measured AUC, accuracy, sensitivity and specificity. As shown in Supplementary Table 5, GLM shows relatively higher performance in all performance metrics.

After collecting the data for all 34 peptides, we identified the best peptide biomarkers to build predictive models. To do this, first, all peptide expressions (abundances) were normalized against peptide expressions of normal controls:


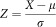


*Z* is the normalized peptide expression, *X* is the peptide expression in each sample, *μ* is the mean of the normal controls and *σ* is the s.d. of the normal controls. We then examined inter-correlation between peptides. To do this, we generated a feature–feature matrix by comparing expression profiles between peptides (Supplementary Fig. 10a). There is low-expression similarity among all tested peptides (median Pearson's *R* is 0.15), suggesting that there is no inter-dependency between tested peptides and they have their own prediction importance. Next, we calculated the importance of each peptide to discriminate two classes (for example, cancer and normal controls) and subsequently selected the top-ranked peptides (from top 3 to top 15). We used these top-ranked peptides as features to build predictive models. To avoid overfitting, 10-fold cross-validation was conducted on a training set. We then examined their prediction performance on a testing set. To obtain stable predictions, predictive models were tested by 100-fold bootstrapping. The resulting average AUC was calculated and used as a performance measure. Sets of top-ranked peptides that show the highest AUC were chosen as the most relevant features. As a result, we selected six peptides for diagnosis and seven peptides for prognosis as the most relevant features (Supplementary Table 6). Peptides that were used as predictors also showed low-expression similarity. Median Pearson's *R* is 0.32 (cancer versus normal controls, Supplementary Fig. 10b) and 0.18 (pT3 versus pT2, Supplementary Fig. 10c).

### Data availability

The raw MS data associated with this manuscript have been submitted to a public repository (the Mass Spectrometry Interactive Virtual Environment; http://massive.ucsd.edu) for others to download. These data are associated with the identifier MassIVE ID MSV000079401 at the FTP site ftp://massive.ucsd.edu/MSV000079401. Cohort B SRM traces for the 34 measured peptides are available through Panorama at the following link (https://panoramaweb.org/labkey/project/EPS_SRM/Cohort%20B%20%28Phase%203%20peptides%29/begin.view?). Skyline exported data for all quantified peptides of cohort B is available in Supplementary Table 7. The authors declare that all other data supporting the findings of this study are available within the article and its Supplementary Information Files.

## Additional information

**How to cite this article:** Kim, Y. *et al.* Targeted proteomics identifies liquid-biopsy signatures for extracapsular prostate cancer. *Nat. Commun.* 7:11906 doi: 10.1038/ncomms11906 (2016).

## Supplementary Material

Supplementary Information Supplementary Figures 1-10

Supplementary Table 1 Cohorts A and B patient information. pGG = pathological Gleason grade; pGGS = pathological Gleason score; ND = not determined; BCR = biochemical recurrence; Mets = metastasis; NED = no evidence of disease

Supplementary Table 2 Performance of predictors with and without additional peptides from the same protein

Supplementary Table 3 Summary of datasets for predictor generation

Supplementary Table 4 Predictor performance depending on training set size*

Supplementary Table 5 Performance of different machine learning algorithms

Supplementary Table 6 Predictor performance of test set depending on the number of biomarker candidates

Supplementary Table 7 Skyline exported data for all peptides quantified in cohort B (34 peptides, 207 samples, technical duplicates, light & heavy peptides = 28152 rows)

## Figures and Tables

**Table 1:** Patient characteristics for EPS urine samples from cohorts A and B.

	***N***	**Mean age±s.e.m.**	**Gleason score range**	**Gleason score (*****n*****)**	**BCR**	**Mets**
*Cohort A*						
Controls	24	59.23±1.49				
Normal	11	57.34±2.09				
BPH	13	60.83±2.08				
Prostate cancer	50	58.9±0.92				
Organ confined	37	57.9±1.04	6–8	6 (12)	0	0
				7 (24)		
				8 (1)		
Extracapsular	13	61.7±1.75	6–9	6 (1)	2	1
				7 (11)		
				9 (1)		
						
*Cohort B*						
Controls	117	60.15±0.77				
Normal	48	58.33±1.36				
BPH	69	61.41±0.87				
Prostate cancer	90	59.72±0.64				
pT2	61	59.43±0.78	6–8	6 (23)	11	3
				7 (37)		
				8 (1)		
pT3	29	60.34±1.15	6–9	6 (2)	10	3
				7 (23)		
				8 (1)		
				9 (2)		

BCR, biochemical recurrence; EPS, expressed prostatic secretion; Mets, metastasis.
