# Supplementary material for: Targeted proteomics identifies liquid-biopsy signatures for extracapsular prostate cancer
Source: Nat Commun. 2016 Jun 28;7:11906. doi: 10.1038/ncomms11906 (PMC4931234; doi:10.1038/ncomms11906)
Supplement: Supplementary Information — Supplementary Figures 1-10 [file ncomms11906-s1.pdf]

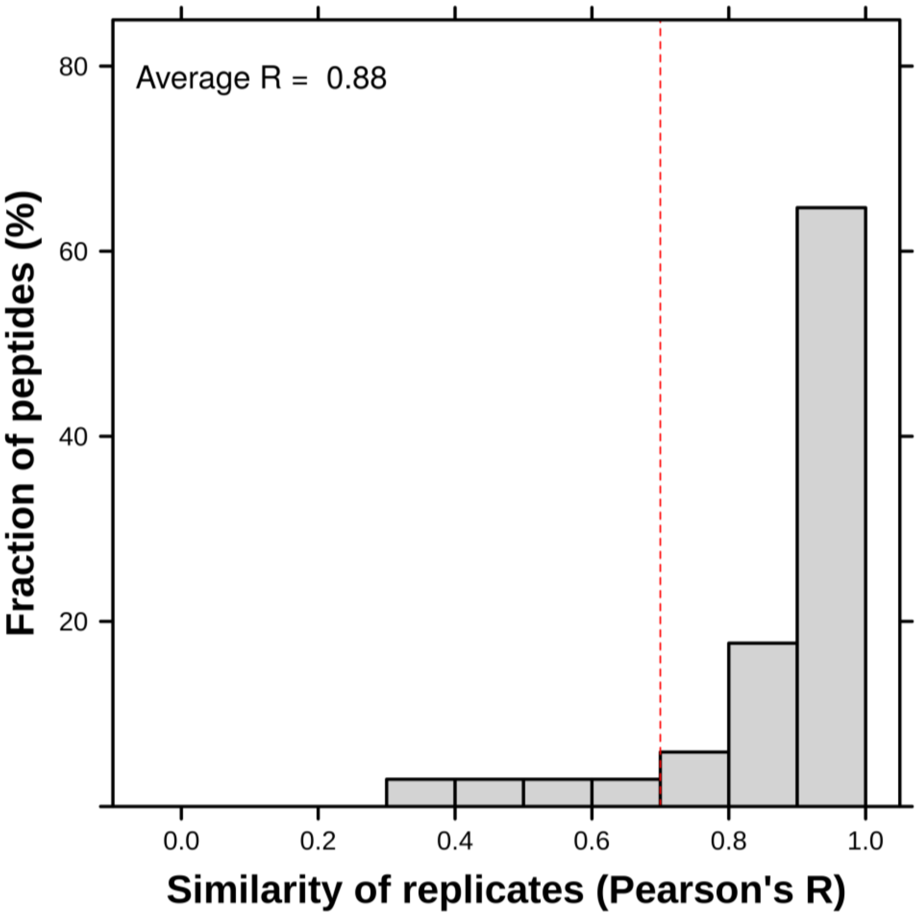

Supplementary Figure 1. Pearson correlation for replicate analyses (all sample types analyzed in duplicate). Representation of the reproducibility (Pearson's correlation) for replicate analyses of all peptides analyzed in Cohort B (n=207). Dotted red line represents samples with high correlation (R>0.7).

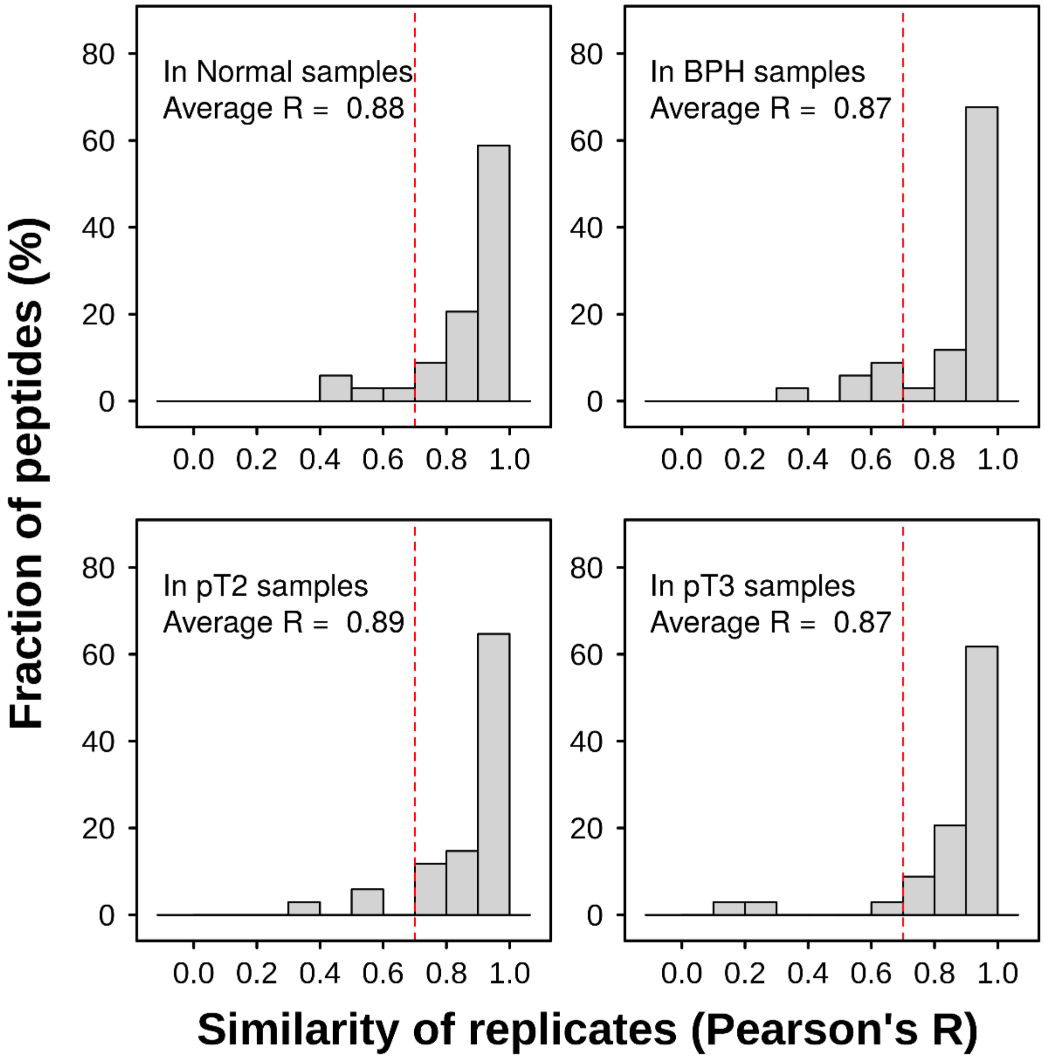

Supplementary Figure 2. Pearson correlation for replicate analyses (individual sample types; risk groups). Representation of the reproducibility (Pearson's correlation) for replicate analyses of all peptides analyzed in Cohort B stratified by patient risk group (normal, BPH, pT2, pT3). Dotted red line represents samples with high correlation (R>0.7).

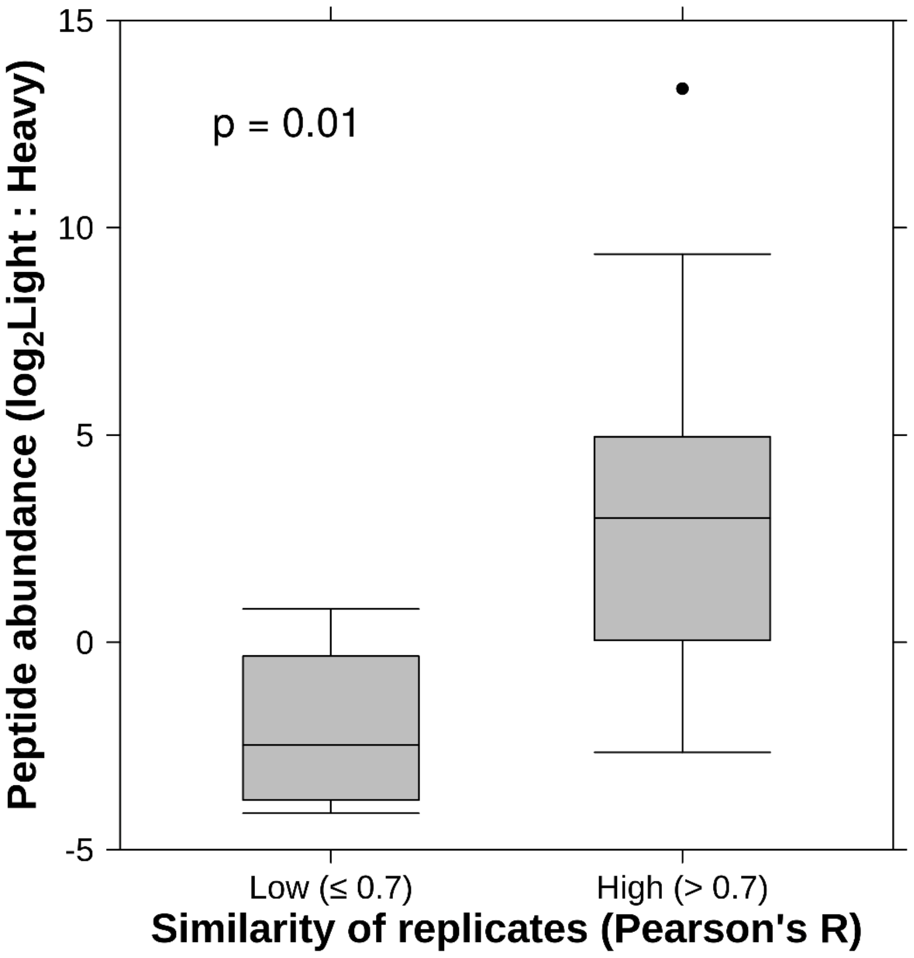

Supplementary Figure 3. Pearson correlation compared by peptide abundance. Comparison of peptide abundance plotted as a function of Pearson's correlation. Peptides with a high concordance between replicate analyses (R>0.7) are significantly more abundant based on SRM quantification.

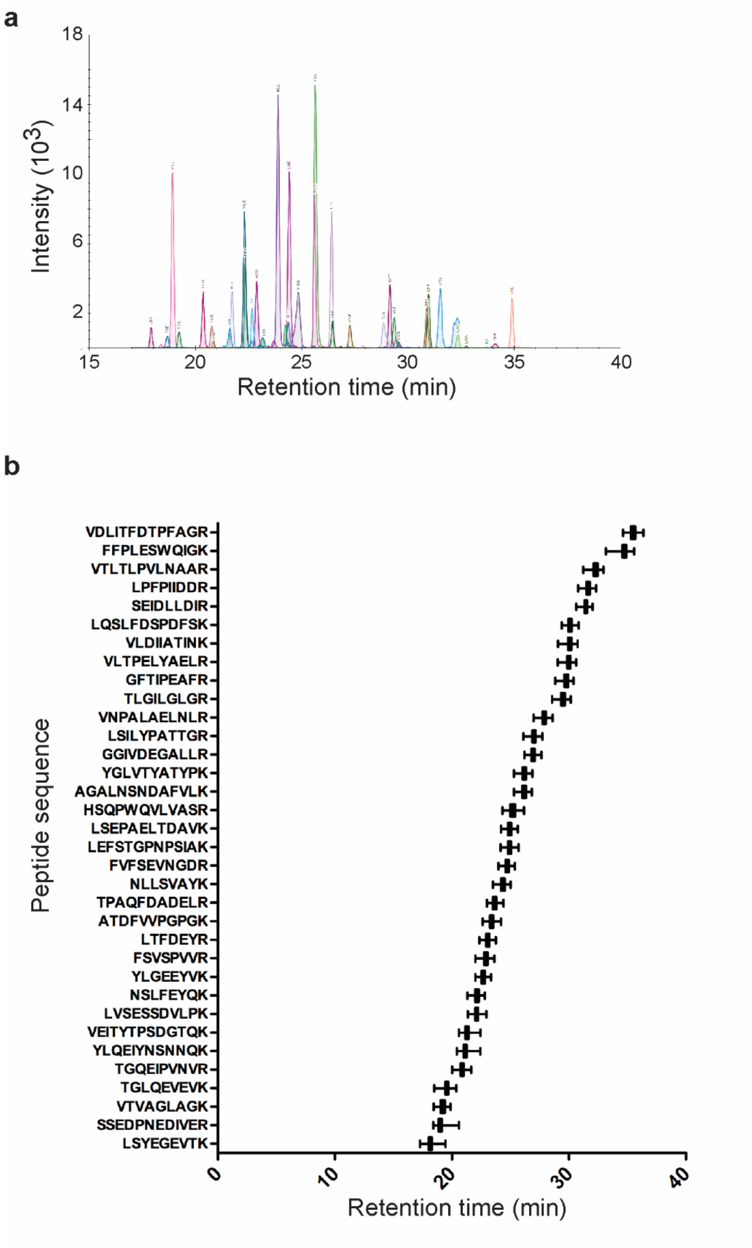

Supplementary Figure 4. Chromatographic retention time of individual peptides. **(a)** Representative chromatogram of the 34 peptides quantified in cohort B. (**b**) The 34 peptides quantified by SRM-MS in all cohort B samples (207 samples analyzed in duplicates; n=414 SRM-MS analyses) demonstrate highly reproducible retention times.

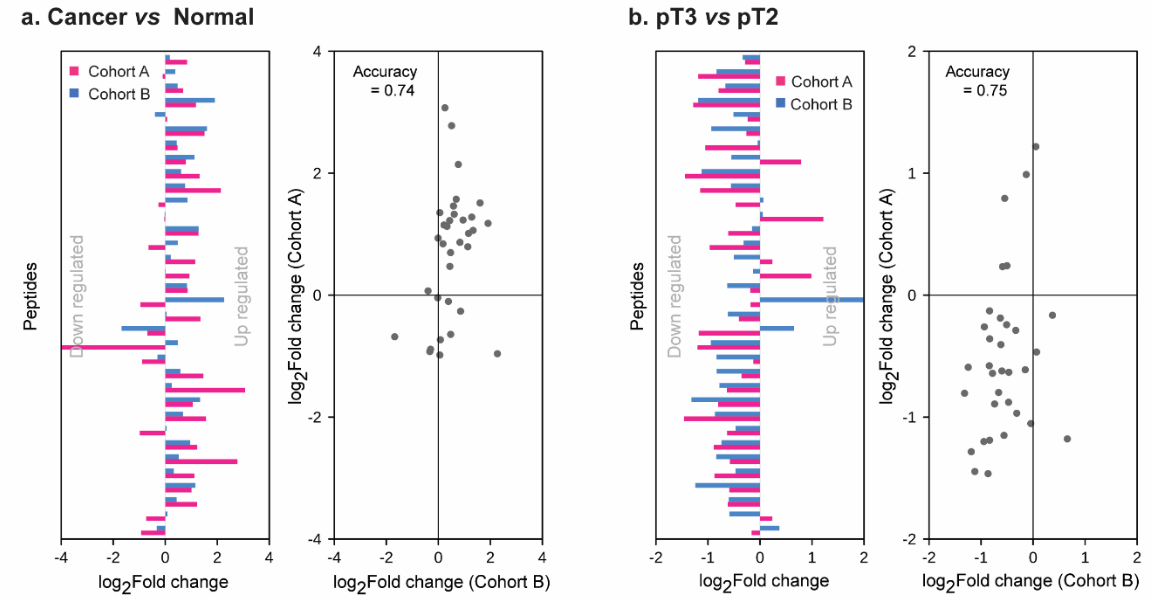

Supplementary Figure 5. Peptide abundance in the two separate patient cohorts. **(a)** Average fold change correlation of cancer *vs.* normal samples for the 34 peptides quantified in cohorts A and B. left side: average fold change (log_2) in both cohorts; rights side: correlation blot. **(b)** Average fold change correlation of pT2 *vs.* pT3 samples for the 34 peptides quantified in cohorts A and B. left side: average fold change (log_2) in both cohorts; rights side: correlation blot.

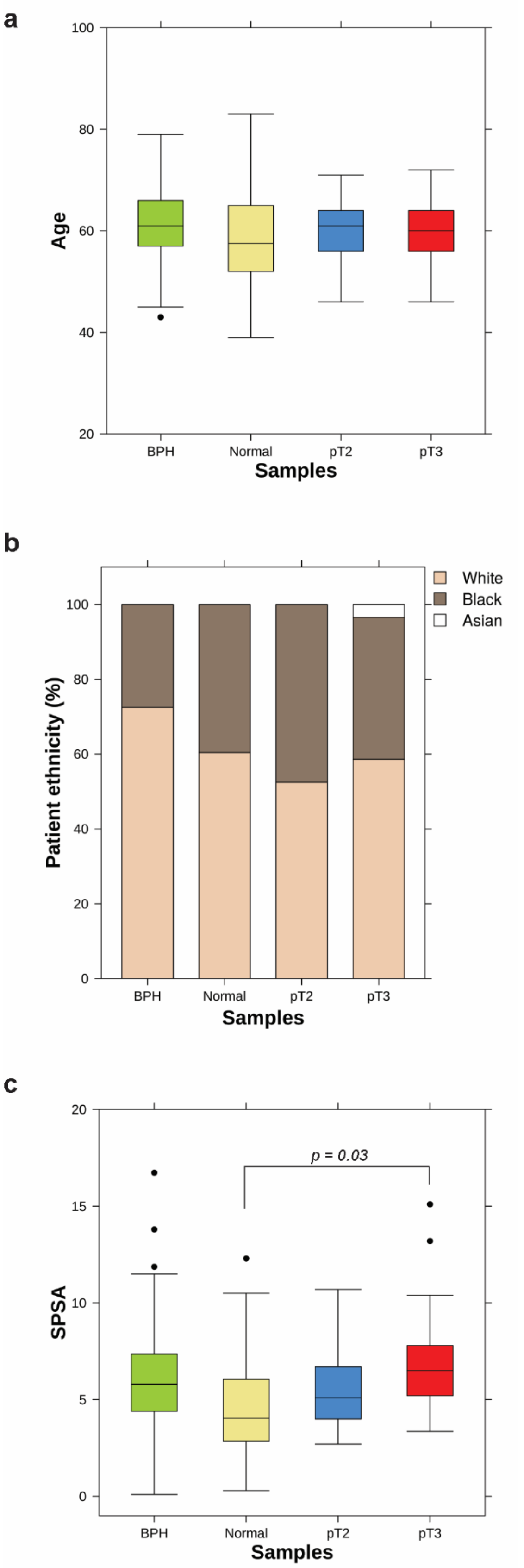

Supplementary Figure 6. Patient characteristics for all cohort B samples (n=207). **(a)** Age distribution; **(b)** Ethnicity; **(c)** Serum PSA distribution (SPSA).

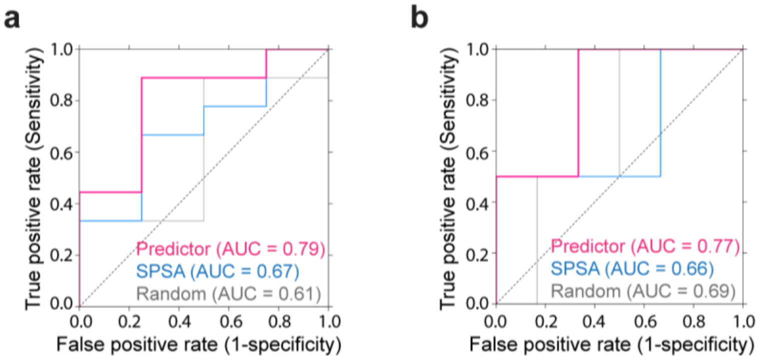

(a)

	Performance result	
	Value	95% CI
AUC	0.79	0.76 - 0.81
Accuracy	0.72	0.69 - 0.74
Sensitivity	0.82	0.79 - 0.84
Specificity	0.49	0.45 - 0.54

(b)

	Performance result	
	Value	95% CI
AUC	0.77	0.74 - 0.80
Accuracy	0.74	0.71 - 0.77
Sensitivity	0.44	0.36 - 0.51
Specificity	0.84	0.80 - 0.87

Supplementary Figure 7. ROC curves for test set analysis. **(a)** Diagnostic signature: the performance for the selected peptide signature (pink), serum PSA alone (blue) and randomly selected peptides (grey) are compared. **(b)** Prognostic signature: the performance for the selected peptide signature (pink), serum PSA alone (blue) and randomly selected peptides (grey) are compared. ROC curves are generated from test set.

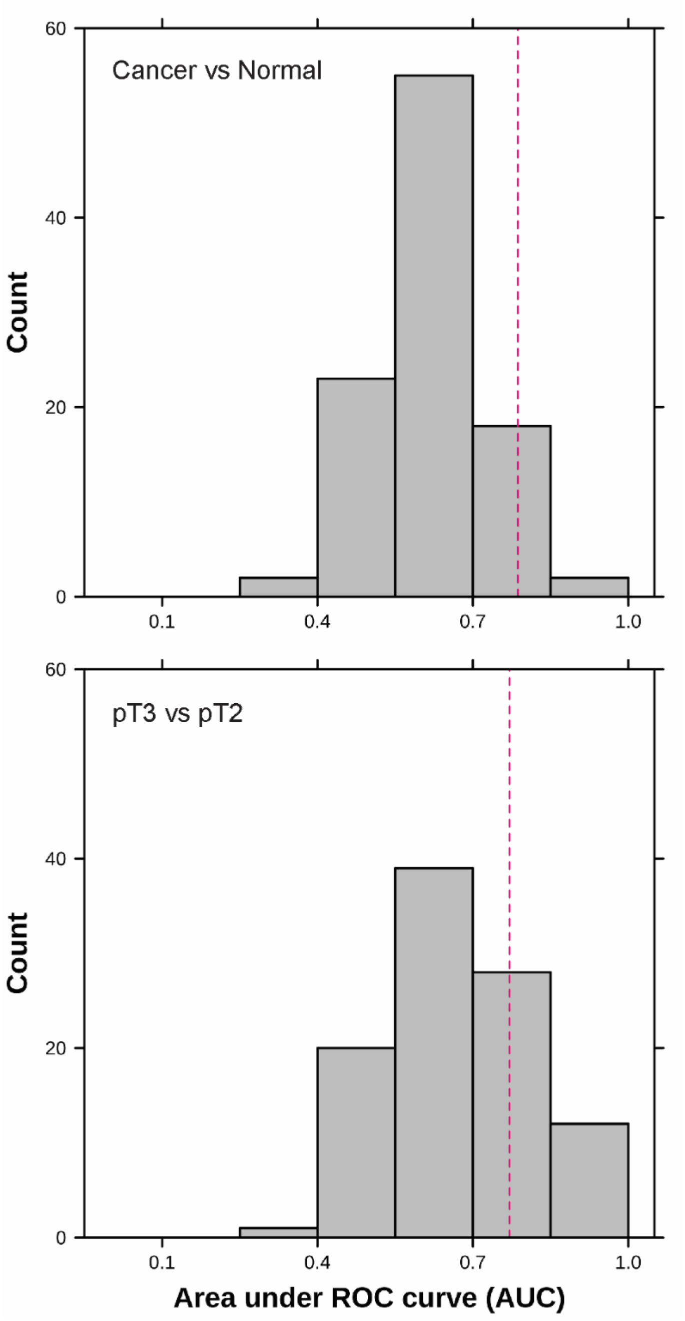

Supplementary Figure 8. Area under the ROC for randomly generated signatures. Distribution of AUCs for randomly selected peptides to generate predictive models for cancer *vs.* normal (top panel) or for pT3 *vs.* pT2 (bottom panel). Pink line indicates AUC for our predictive models based on identified peptides. AUCs are measured from test set.

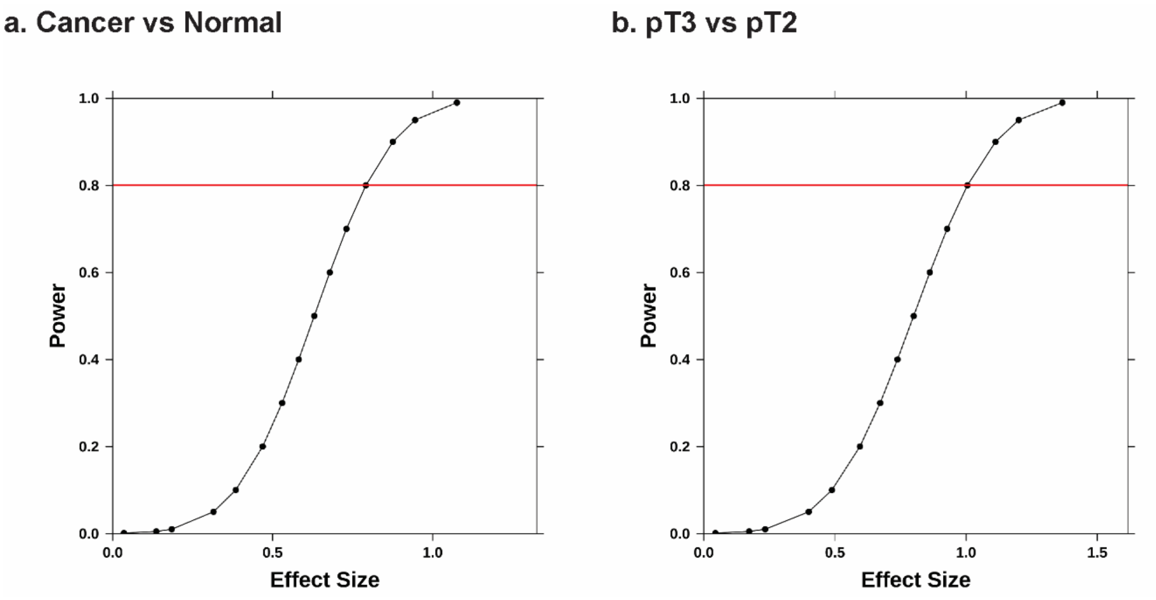

Supplementary Figure 9. Power analyses for all analyzed samples to distinguish indicated patient risk groups.

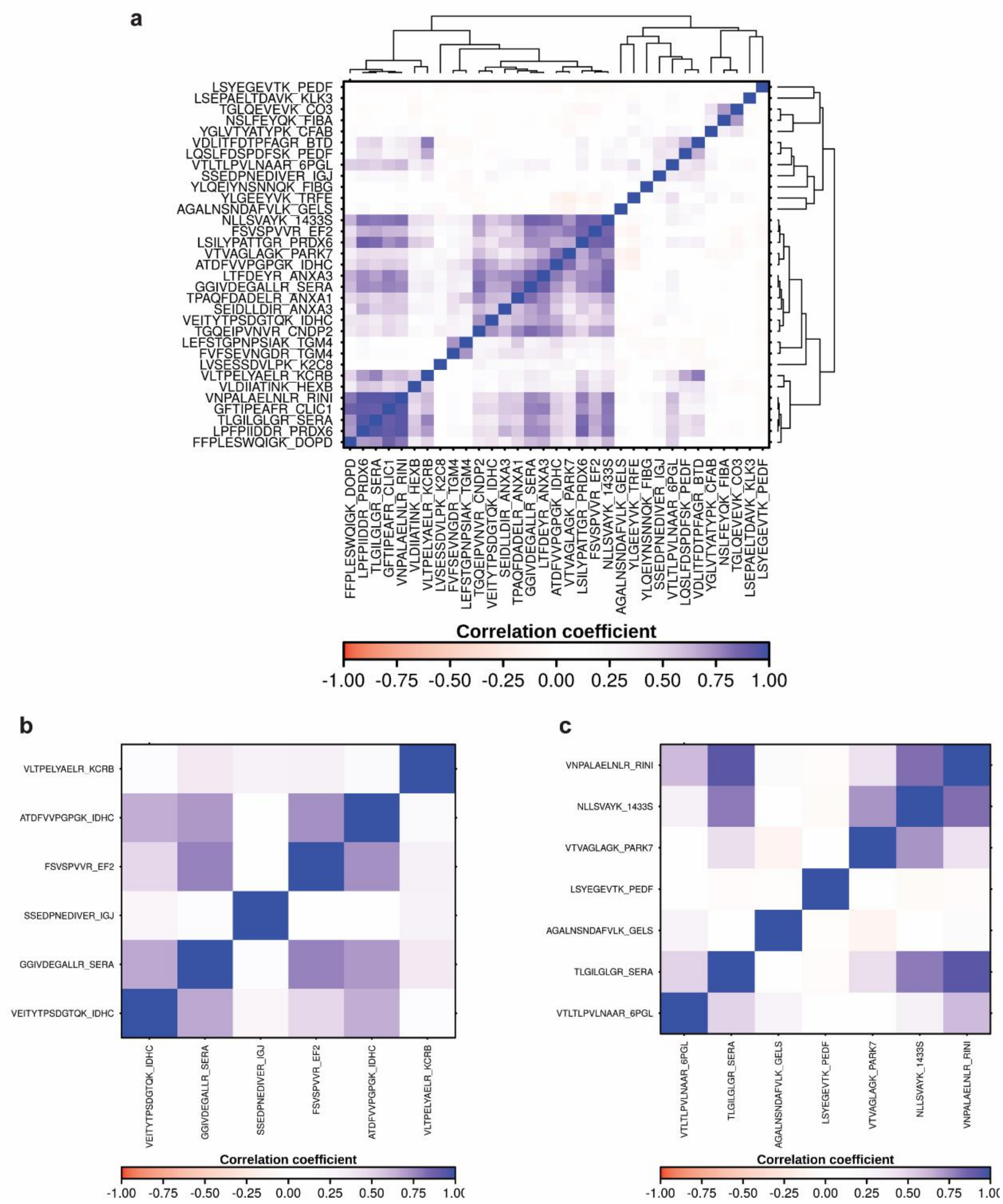

Supplementary Figure 10. Inter-correlation between peptides. **(a)** Correlation matrix of peptide-peptide expression. Expression profiles between all 34 peptides quantified in cohort B are compared using Pearson's correlation coefficient (R). Peptide-peptide matrix comparing the quantitative expression profiles of **(b)** the 6 diagnostic signature peptides and **(c)** the 7 prognostic signature peptides.
